# Efficacy and safety of angiogenesis inhibitors plus immune checkpoint inhibitors in advanced soft tissue sarcoma: a real-world, single-center study

**DOI:** 10.1038/s41598-023-30412-6

**Published:** 2023-02-28

**Authors:** Zengjun Liu, Jing Xu, Mengyao Liu, Wenyu Hu, Ni Xu, Dongyuan Zhu

**Affiliations:** Rare Tumors Department, Shandong Cancer Hospital and Institute, Shandong First Medical University and Shandong Academy of Medical Sciences, 440 Jiyan Road, Jinan, 250117 Shandong China

**Keywords:** Cancer, Oncology

## Abstract

Angiogenesis inhibitors (AIs) and immune checkpoint inhibitors (ICIs) are new treatment options for advanced soft tissue sarcoma (STS) patients. This study evaluated the efficacy and safety of AIs plus ICIs in patients with advanced STS. A retrospective cohort study was performed on STS patients treated with AIs and ICIs at Shandong Cancer Hospital and Institute between August 2020 and December 2021. The primary endpoint was objective response rate (ORR); secondary endpoints included progression-free survival (PFS), disease control rate (DCR), overall survival (OS), and adverse events. Thirty-three patients were enrolled; 27 were evaluable for objective response. The ORR and DCR were 48.1% (95% CI 30.7–66.0%) and 85.2% (95% CI 67.5–94.1%). With a median follow-up of 7.6 months (range, 0.8–25.5), the median PFS for all 33 patients was 8.90 months (95% CI 5.98–11.82). The median OS was not reached. The most common treatment-related adverse events (TRAEs) of any grade were hypertension (50.0%), ECG T-wave abnormality (30.0%), hypothyroidism (26.7%), elevated alanine aminotransferase or aspartate aminotransferase (23.3%), elevated thyroid-stimulating hormone (23.3%), and fatigue (16.7%). The most common grade 3–4 TRAE was hypertension (27.3%). Three serious TRAEs (two myocarditis and one rapid atrial fibrillation) were recorded. This study suggests that adding AIs to ICIs is beneficial in STS.

## Introduction

Soft tissue sarcoma (STS) is a rare, heterogeneous group of mesenchymal malignancies, with more than 70 subtypes characterized by distinct morphological and genetic features. Anthracycline-based chemotherapy is the standard treatment for most patients with advanced STS^1,2^.

For patients with STS who failed first-line treatment, angiogenesis inhibitors (AIs) and immune checkpoint inhibitors (ICIs) have emerged as new treatment options^2^. Anlotinib, an oral multi-target tyrosine kinase inhibitor that exerts significant anti-angiogenesis effects, has been approved to treat STS in China^3^. In a single-arm, phase 2 trial (SARC028), pembrolizumab showed activity in patients with STS, especially in patients with certain subtypes, such as undifferentiated pleomorphic sarcoma (UPS) and de-differentiated liposarcoma (DDLPS)^4^. In another phase 2 trial, nivolumab combined with ipilimumab demonstrated promising efficacy in certain sarcoma subtypes, including UPS, myxofibrosarcoma, and angiosarcoma^5^. Meanwhile, combination therapy with AIs and ICIs has been tested across various solid tumours. Increasing evidence has shown that AIs and ICIs complement each other and exhibit synergistic anti-tumour effects^6,7^. Several studies using the combination regimes of axitinib plus pembrolizumab, sunitinib plus nivolumab, and anlotinib plus TQB2450 (a novel programmed death ligand-1 inhibitor), respectively, have demonstrated the feasibility of this promising treatment strategy for STS^8–10^.

However, considering the heterogeneity of STS, it is necessary to evaluate the strategy in more subtypes further. Thus, we designed a retrospective study to evaluate the efficacy and safety of combination therapy with AIs and ICIs in a real-world setting.

## Results

### Patient characteristics

**Figure 1:**
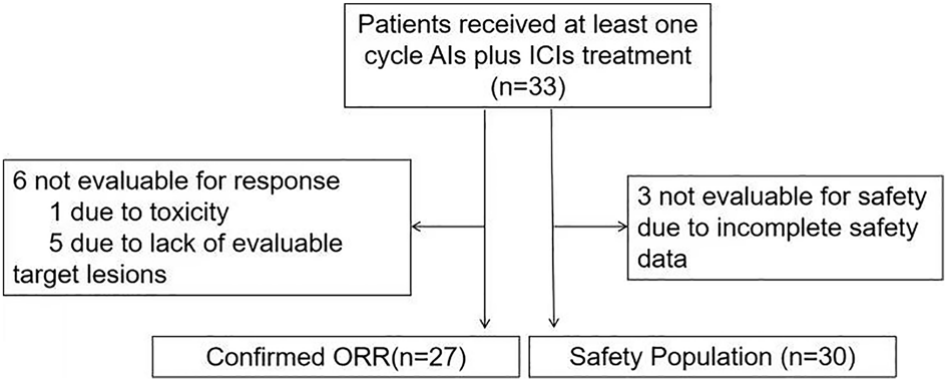
Study profile.

A total of 33 patients were included in this study (Fig. 1). The baseline characteristics of the patients are depicted in Table 1 and Supplementary Table S1. Fifteen (45.5%) patients were male, and 18 (54.5%) were female. The median age of all patients was 56 years (range: 7–87 years). The most common histological subtype was alveolar soft part sarcoma (ASPS, 10 cases, 30.3%), followed by DDLPS (4 cases, 12.1%), UPS (3 cases, 9.1%), undifferentiated sarcoma (US, 2 cases, 6.1%), leiomyosarcoma (LMS, 2 cases, 6.1%), clear cell sarcoma (CCS, 2 cases, 6.1%), angiosarcoma (AS, 2 cases, 6.1%), inflammatory myofibroblastic tumour (IMT, 2 cases, 6.1%), synovial sarcoma (SS, 1 case, 3.0%), myofibroblastic sarcoma (1 case, 3.0%), spindle cell sarcoma (1 case, 3.0%), myxofibrosarcoma (1 case, 3.0%), rhabdomyosarcoma (1 case, 3.0%), and malignant peripheral nerve sheath tumour (1 case, 3.0%). Fifteen (45.5%) patients were treated as first-line therapy; 25 (75.8%) patients had a surgical history. All were R0 surgery.

**Table 1:** Baseline characteristics.

Patient characteristics	N = 33
Age (years), median (range)	56 (range: 7–87)
Sex, n (%)	
Male	15 (45.5)
Female	18 (54.5)
Histology, n (%)	
Alveolar soft part sarcoma	10 (30.3)
De-differentiated liposarcoma	4 (12.1)
Undifferentiated pleomorphic sarcoma	3 (9.1)
Undifferentiated sarcoma	2 (6.1)
Leiomyosarcoma	2 (6.1)
Clear cell sarcoma	2 (6.1)
Angiosarcoma	2 (6.1)
Inflammatory myofibroblastic tumour	2 (6.1)
Synovial sarcoma	1 (3.3)
Myofibroblastic sarcoma	1 (3.3)
Spindle cell sarcoma	1 (3.3)
Myxofibrosarcoma	1 (3.3)
Rhabdomyosarcoma	1 (3.3)
Malignant peripheral nerve sheath tumour	1 (3.3)
Primary tumour location, n (%)	
Extremities	16 (48.5)
Peritoneal and retroperitoneal cavity	12 (36.4)
Visceral	4 (12.1)
Head	1 (3.0)
Surgical history, n (%)	
Yes	25 (75.8)
No	8 (24.2)
Previous chemotherapy, n (%)	
Yes	15 (45.4)
No	18 (54.5)
Previous radiotherapy, n (%)	
Yes	5 (15.2)
No	28 (84.8)
Treatment lines of AIs plus ICIs, n (%)	
First-line	15 (45.5)
Second-line and higher	18 (54.5)

*AIs* Angiogenesis inhibitors, *ICIs* Immune checkpoint inhibitors.

### Efficacy

**Figure 2:**
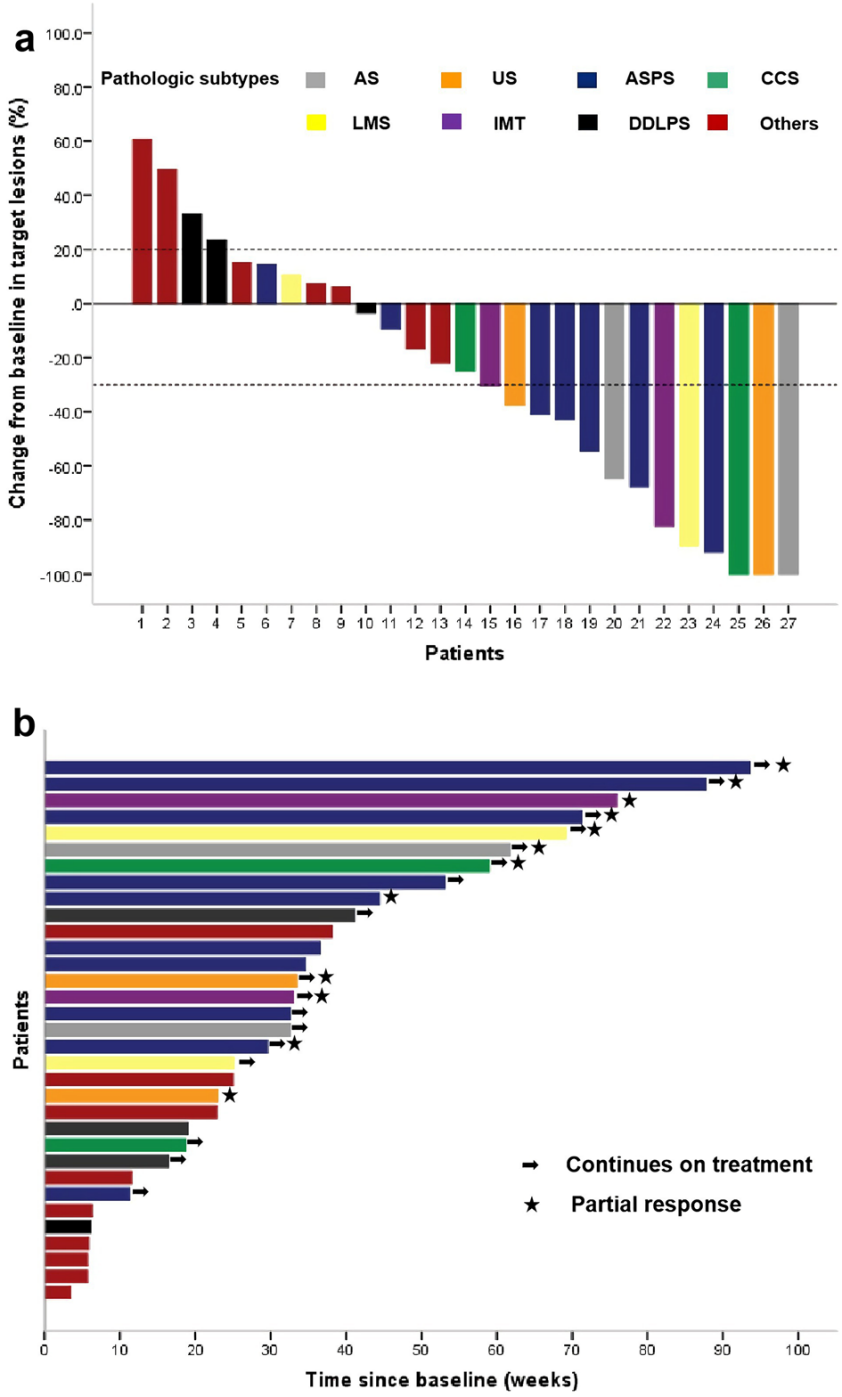
(**a**) Waterfall plot of the best percentage change from baseline in target lesions in 27 evaluable patients. AS, Angiosarcoma; US, Undifferentiated sarcoma; ASPS, Alveolar soft-part sarcoma; CCS, Clear cell sarcoma; LMS, Leiomyosarcoma; IMT, Inflammatory Myofibroblastic Tumor; DDLPS, De-differentiated liposarcoma; Others include rhabdomyosarcoma (n = 1), myxofibrosarcoma (n = 1), undifferentiated pleomorphic sarcoma (n = 1), spindle cell sarcoma (n = 1), malignant peripheral nerve sheath tumour (n = 1), myofibroblastic sarcoma (n=1) and synovial sarcoma (n = 1). (**b**) Progression-free survival of patients. Each patient is represented by a horizontal bar (n = 33). The stars represent patients achieving objective responses. The arrows represent patients non-progressing during the last radiological assessment.

**Figure 3:**
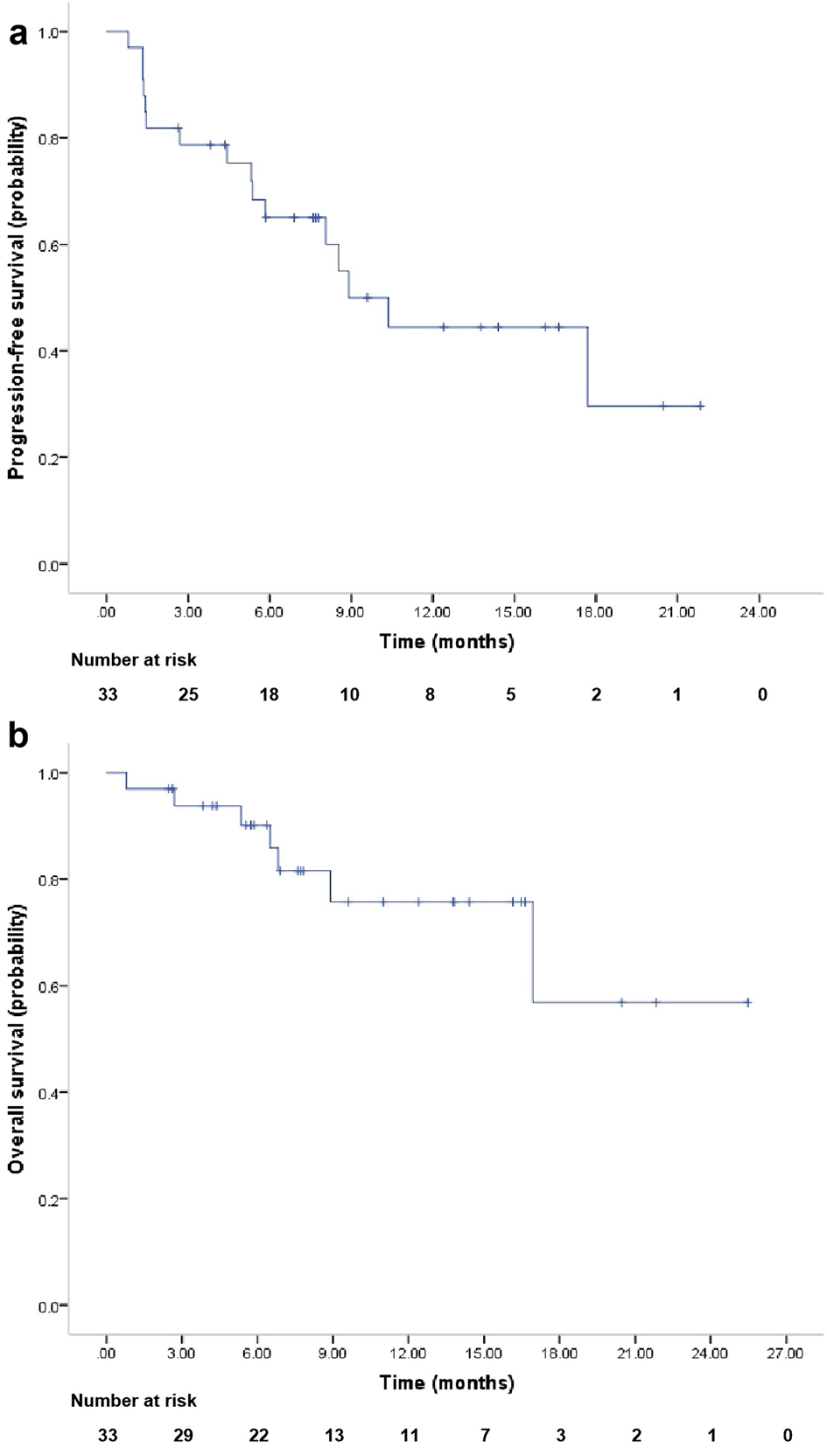
Kaplan–Meier curves for (**a**) progression-free survival and (**b**) overall survival in all 33 patients.

The last follow-up date of this study was 1st October 2022. The median follow-up duration for the 33 patients from treatment to the date of data cut-off was 7.6 months (range, 0.8–25.5 months). The median number of cycles administered was 8 (range, 1–29). Of the 27 patients evaluable for objective response, 3 (11.1%) achieved complete response (CR), 10 (37.0%) achieved partial response (PR), and 10 (37.0%) achieved stable disease (SD); the objective response rate (ORR) and disease control rate (DCR) was 48.2% (95% confidence interval (CI), 30.7–66.0%) and 85.2% (95% CI 67.5–94.1%), respectively. The best percentage change from the baseline of target lesions is shown in Fig. 2a. The progression-free survival (PFS) of the patients is depicted in Fig. 2b. The median PFS for all 33 patients was 8.90 months (95% CI 5.98–11.82, Fig. 3a). The median overall survival (OS) was not reached (Fig. 3b).

Of the seven ASPS patients evaluable for objective response, 5 (71.4%) achieved PR, and 2 (28.6%) achieved SD, so the DCR was 100%. No patient achieved CR. Of the 20 non-ASPS patients evaluable for objective response, 3 (15.0%) achieved CR, 5 (25.0%) achieved PR, and 8 (40.0%) achieved SD, so the DCR was 90%.

### Safety

All 30 evaluable patients experienced treatment-related adverse events (TRAEs, Table 2). The most common TRAEs of any grade included hypertension (50.0%), ECG T-wave abnormality (30.0%), hypothyroidism (26.7%), elevated alanine aminotransferase or aspartate aminotransferase (23.3%), elevated thyroid-stimulating hormone (23.3%), and fatigue (16.7%). Grade 3–4 TRAEs were observed in 50% of patients, and the most common grade 3–4 TRAE was hypertension (27.3%). Three serious TRAEs were recorded, including two cases of myocarditis and one case of rapid atrial fibrillation. No unexpected TRAEs were observed. No treatment-related deaths occurred.

**Table 2:** Toxicity profile of 30 evaluable patients.

Toxicity	Any grade, n (%)	≥ Grade 3, n (%)
Hypertension	15 (50.0)	9 (27.3)
ECG T-wave abnormality	9 (30.0)	–
Hypothyroidism	8 (26.7)	2 (6.7)
Elevated alanine aminotransferase or aspartate aminotransferase	7 (23.3)	1 (3.3)
Elevated thyroid-stimulating hormone	7 (23.3)	–
Fatigue	5 (16.7)	–
Elevated γ-glutamyltransferase	4 (13.3)	–
Hyperbiliubinemia	4 (13.3)	–
Hand-foot syndrome	3 (10.0)	–
Hypertriglyceridemia	3 (10.0)	–
Hyperthyroidism	3 (10.0)	–
Elevated B-type natriuretic peptide	3 (10.0)	1 (3.3)
Elevated alkaline phosphatase	3 (10.0)	–
Hyperuricemia	3 (10.0)	–
Leukopenia	3 (10.0)	–
Sinus bradycardia	3 (10.0)	–
Odynophagia	2 (3.3)	–
Lymphopenia	2 (6.7)	–
Hypercholesterolemia	2 (6.7)	–
Diarrhea	2 (6.7)	–
Elevated Troponin T	2 (6.7)	–
Ventricular arrhythmia	2 (6.7)	–
Neutropenia	2 (6.7)	–
Gastrointestinal bleeding	1 (3.3)	–
Hypoalbuminemia	1 (3.3)	–
Oral mucositis	1 (3.3)	–
Nausea or vomiting	1 (3.3)	–
Rash and pruritis	1 (3.3)	1 (3.3)
Swollen gums	1 (3.3)	–
Hyponatremia	1 (3.3)	–
Hypophosphatemia	1 (3.3)	–
Hypoferremia	1 (3.3)	–
Thrombocytopenia	1 (3.3)	–
Anaemia	1 (3.3)	1 (3.3)
Sinus tachycardia	1 (3.3)	–

## Discussion

Accumulated pre-clinical evidence has shown that AIs enhance the efficacy of ICIs by normalizing abnormal tumour vessels and increasing the infiltration of immune effector cells into tumours^7^. In animal models, lenvatinib, a multiple receptor tyrosine kinase inhibitor, was demonstrated to reduce tumour-associated macrophages and increase the percentage of activated CD8+ T cells secreting interferon (IFN)-γ + and granzyme B (GzmB). The addition of an anti–PD-1 antibody further increased the percentage of CD8+ T cells via the IFN signaling pathway, which provides a scientific rationale for combination therapy of lenvatinib with PD-1 blockade to improve cancer immunotherapy^11^. Our study shows that AIs plus ICIs demonstrated promising clinical activity in STS, with an ORR of 48.1% (13/27) and a median PFS of 8.9 months.

The SARC028 study reported a 40% (4/10) ORR to pembrolizumab monotherapy in patients with UPS^4^. In the Alliance A091401 study, a confirmed response was seen in 33.3% (2/6) of patients with UPS in the nivolumab plus ipilimumab cohort^5^. In a recently reported single-center phase 2 trial evaluating durvalumab, an anti-PD-L1 drug, and tremelimumab, an anti-CTLA-4 drug, in advanced or metastatic soft tissue and bone sarcomas, 20% (1/5) of patients with UPS showed an objective response^12^. However, notably, in several previous phase 2 trials reporting the efficacy of AIs plus ICIs among patients with UPS, the ORR was 0 (0 of 5) to axitinib plus pembrolizumab treatment, 0 (0 of 6) to nivolumab plus sunitinib treatment, and 0 (0 of 5) to anlotinib plus TQB2450 treatment^8–10^. Further evidence is needed to confirm the large gap and explain the underlying mechanism.

ASPS is a rare STS that is not responsive to chemotherapy. However, molecular studies have demonstrated the co-expression of VEGFR, PDGFRB, RET, and MET and high PD-L1 expression in ASPS cells, which predict the favorable treatment response of multi-target AIs and ICIs^8,13^. In a phase 2 trial of anlotinib for the treatment of advanced STS, 23.7% (9 of 38) of patients with ASPS achieved a partial response, and the median PFS was 18.23 months^14^. In another phase 2 trial of atezolizumab in ASPS, the ORR was 37.2% (16 of 43), and the median treatment duration was 11.3 months^15^. In several phase 2 trials reporting the efficacy of AIs plus ICIs among patients with ASPS, the ORR was 54.5% (6 of 11) to axitinib plus pembrolizumab treatment, 57% (4 of 7) to nivolumab plus sunitinib treatment, and 75% (9 of 12) to anlotinib plus TQB2450 treatment, which suggested superior efficacy of combination therapy over monotherapy with either AIs or ICIs^8–10^. In the present study, 71.4% (5/7) of patients with ASPS displayed a partial response, confirming the favorable efficacy of the combination therapy in this subtype.

AS is a rare type of cancer that develops in the inner lining of blood vessels and lymph vessels. In a phase 2 study including 40 patients with AS, sorafenib yielded a 13.5% (5/37) response rate, a median PFS of 3.8 months, and a median OS of 14.9 months^16^. Pazopanib also showed a 20% (8/40) response rate, median PFS of 3 months, and median OS of 9.9 months in a retrospective study of advanced vascular sarcomas^17^. In the Alliance A091401 study, a confirmed response was seen in 33.3% (1/3) of patients with AS in the nivolumab plus ipilimumab cohort^5^. In the phase 2 trial evaluating the durvalumab plus tremelimumab in advanced or metastatic soft tissue and bone sarcomas, 20% (1/5) of patients with AS showed objective response^12^. In the phase 2 trial reporting the efficacy of nivolumab plus sunitinib treatment among patients with AS, the ORR was 42.9% (3/7)^9^. In the present study, two patients with AS showed an objective response. All the above studies confirmed the efficacy of combination therapy and monotherapy with either AIs or ICIs in this specific subtype.

The SARC028 study reported 20% (2/10) ORR to pembrolizumab monotherapy in patients with DDLPS^4^. Correlative analyses of the SARC028 trial revealed an association between immune infiltrate and response to pembrolizumab in DDLPS^18^. In a retrospective study from China, 36.3% (4/11) of patients with DDLPS showed an objective response to AIs plus ICIs combination therapy^19^. In our study, only three patients with DDLPS were evaluable for ORR, and none showed an objective response. Efforts to confirm the activity of AIs plus ICIs in an expansion cohort of DDLPS patients are needed.

AIs and ICIs have also been explored as therapeutic modalities in LMS. Although sunitinib and sorafenib showed no benefit in LMS, pazopanib demonstrated modest efficacy (ORR, 11%; PFS, 3 months; OS, 17.5 months) in patients with uterine LMS in a retrospective analysis based on two clinical trials^16,20,21^. In the subsequent randomized, placebo-controlled clinical trials of anlotinib and regorafenib, a significant increase in PFS compared with placebo (5.83 months vs. 1.43 months, 3.7 months vs. 1.8 months, respectively) in patients with LMS was demonstrated, which confirmed the therapeutic effect of AIs in patients with LMS^14,22^. In contrast, none of the patients with LMS in the SARC028 study showed a response to pembrolizumab, which is consistent with a phase 2 evaluation of nivolumab that was stopped early for futility, suggesting that single-agent ICIs therapy may not be able to elicit a response in LMS^4,23^. However, in the Alliance A091401 study, 16% of patients with LMS in the nivolumab plus ipilimumab arm showed an objective response, which renewed hope for ICIs in patients with LMS^5^. In two phase 2 trials reporting the efficacy of AIs plus ICIs among patients with LMS, the ORR was 16.7% (1/6) to axitinib plus pembrolizumab treatment and 25% (1/4) to anlotinib plus TQB2450 treatment. In the present study, one of the two patients with LMS showed an objective response. In another retrospective study from China with a larger sample size, 19% (4/21) of patients with LMS showed an objective response to AIs plus ICIs combination therapy, suggesting the modest efficacy of the combination regime in patients with LMS^19^.

The toxicity profile of AIs plus ICIs therapy is consistent with previous studies of the drugs as monotherapy. No unexpected TRAEs occurred. The main ≥ grade 3 TRAE was hypertension, which was manageable with antihypertensive drugs. Two patients who experienced myocarditis recovered after the administration of steroids. One patient experienced rapid atrial fibrillation, which was controlled with β-blockers. All three cases with serious TRAEs stopped AIs plus ICIs therapy permanently.

The present study has some drawbacks. First, this is a retrospective study with a small sample size; hence, selection bias cannot be avoided. Second, the irregular radiology examination in clinical practice may lead to bias in evaluating PFS.

In conclusion, our study suggests that adding AIs to ICIs shows encouraging benefits in STS. The safety profiles of the combination strategy are manageable. Additional studies with larger sample sizes and controlled arms are needed.

## Methods

### Patients

We reviewed all patients with STS treated at Shandong Cancer Hospital and Institute between 1st August 2020 and 17th December 2021. The main inclusion criteria were: (a) histological diagnosis of STS; (b) patients were administered with AIs plus ICIs combination therapy. We excluded patients who received prior treatment with any AI or ICI. The study profile of this study is illustrated in Fig. 1. This retrospective analysis was approved by the Ethics Committee of Shandong Cancer Hospital and Institute. The ethics review committee waived the requirement for informed consent from patients. All methods were performed in accordance with the relevant guidelines and regulations.

### Treatment and follow-up

The AIs included anlotinib, apatinib, and sunitinib. The ICIs included sintilimab, toripalimab, camrelizumab, and pembrolizumab. The treatment regimens are listed in Supplementary Table S1. Follow-up was routinely conducted every three months.

### Evaluation of efficacy and adverse events

The following data were reviewed: patients’ demographic characteristics, histological subtype, radiological imaging, laboratory test results, survival data, and adverse events (AEs) during the treatment based on the NCI Common Terminology Criteria for Adverse Events (NCI CTCAE) v5.0 criteria. The tumour response was assessed every two cycles according to the Response Evaluation Criteria in Solid Tumours (RECIST) version 1.1. The primary endpoint was ORR; the secondary endpoints included PFS, DCR, OS, and AEs. ORR was defined as the proportion of patients who achieved complete response (CR) or partial response (PR). DCR was defined as the proportion of patients whose best response was CR, PR, or SD. PFS was defined as the time from the initiation of treatment to the time of disease progression or death due to any cause, whichever occurred first. OS was measured from the initiation of treatment to the date of death from any cause. Patients who were event-free or lost to follow-up were censored at the time of the last visit.

### Statistical analysis

All statistical analyses were performed with SPSS 21.0 statistical software. Continuous data were presented as mean and standard deviation if normally distributed or median and range. Categorical data were presented as numbers and percentages. PFS and OS were estimated using the Kaplan–Meier survival method.

## Supplementary Information

Supplementary Information.

## Data Availability

Supplemental material for this article is available online. Further enquiries can be directed to the corresponding author.
